# Soil characteristics associated with historical commensal rodent plague foci in Yunnan Province, China

**DOI:** 10.3389/fvets.2026.1797277

**Published:** 2026-05-14

**Authors:** Zhi-Qiong Ai, Rui Li, Yao Zhang, Li Wu, Yun-Yan Luo, Ru-Dan Hong, Jia-Xiang Yin

**Affiliations:** 1 School of Public Health, Dali University, Dali, Yunnan, China; 2 Jining Center for Disease Control and Prevention, Jining, Shandong, China

**Keywords:** logistic regression, metal elements, natural plague foci, plague natural foci, principal component analysis, soil characteristics, surface soil, Yunnan

## Abstract

**Background:**

Soil may influence pathogen persistence and outbreak dynamics, yet its characteristics and potential association with historical plague occurrence remain poorly understood. This study aimed to characterize the surface soil properties of three commensal rodent plague foci in Yunnan Province, China, and to explore their site-specific association with historical plague village status.

**Methods:**

From July to August 2019, 230 soil samples were collected from paired historical plague (*n* = 108) and non-plague (*n* = 122) villages in Mile, Mangshi, and Lianghe counties. Soil pH, soil electrical conductivity (SEC), soil organic matter (SOM), and seven metal elements (Fe, Ca, Ti, Co, Cu, Ni, V) were analyzed. Non-parametric tests, Spearman correlation, principal component analysis (PCA), and binary logistic regression were employed for data analysis.

**Results:**

The soils were overall acidic (median pH 5.82), slightly salinized (median SEC 166.51 μS/cm), with moderate SOM (median 21.14 g/kg). Concentrations of Fe, Ti, Co, Cu, Ni, and V significantly exceeded national background values (all *p* < 0.001). Pronounced regional differences (*p* < 0.001) were observed across the three sites: in Mile, soils were near-neutral (median pH 6.79), with the highest SEC (207.5 μS/cm) and the highest concentrations of Fe (91741.03 mg/kg), Cu (79.94 mg/kg), and V (310.1 mg/kg), but the lowest SOM (20.5 g/kg); in Mangshi, soils were acidic (median pH 5.57), with intermediate SEC (165.9 μS/cm) and metal levels, and moderate SOM (21.41 g/kg); in Lianghe (*n* = 71): Soils were the most acidic (median pH 5.48), with the lowest SEC (147.1 μS/cm) and lowest metal concentrations, but the highest SOM (22.05 g/kg). *Post-hoc* comparisons confirmed that Mile had significantly higher pH, SEC, Fe, Ti, Co, Cu, Ni, and V than both Mangshi and Lianghe (Bonferroni-corrected *p* < 0.017), while no significant differences in SOM were found across regions (*p* = 0.634). At the provincial level, historical plague villages had significantly lower Ni and V than non-plague villages (both *p* < 0.05), though regional sub-analyses showed that this pattern was not consistent across all foci, highlighting context-dependent variations. Multivariate analysis extracted four principal components (cumulative variance: 83.72%). Multivariate logistic regression suggested that the heavy metal-rich principal component (PC1) was independently associated with reduced likelihood of historical plague village classification, while loam texture was associated with lower odds of historical plague occurrence relative to sandy soil.

**Conclusion:**

Soils in the three plague foci are acidic, slightly saline, and enriched in several metals, with properties varying significantly by region. In this exploratory, cross-sectional analysis, a multivariate soil profile dominated by heavy metals and loam texture showed a statistical association with historical plague occurrence. These site-specific findings provide crucial baseline data and a multivariate analytical framework for future investigations into the complex, and likely context-dependent, relationships between soil environments and plague ecology.

## 1 Introduction

Plague is a fatal zoonotic disease characterized by alternating periods of quiescent and epizootics. As a typical natural focal disease transmissible to humans, it remains a persistent threat to public health. The disease is caused by *Yersinia pestis* (*Y. pestis*), which is maintained in wild rodent populations and transmitted by fleas. Human plague presents three main clinical forms: pneumonic, bubonic, and septicemic plague, which may occur sporadically, in outbreaks, or as large epidemics (1). Over the past 2,000 years, several major pandemics have caused hundreds of millions of deaths, and natural reservoirs of *Y. pestis* remain globally, including in Yunnan Province, China. In China, human plague cases rose from 16 in 1999 to 254 in 2000. Although incidence has since declined, the case fatality rate has increased 2.5-fold, reaching 100% in some severe areas, and animal plague activity has fluctuated widely (2). In Yunnan Province, after a 10-year quiescent period, human plague re-emerged in 2016 with one primary septicemic case and one bubonic case, and animal plague activity was reported in Xishuangbanna Prefecture in 2020 (3).

Historically, at least three major global plague pandemics have been recorded: the First (or Justinian) Plague Pandemic (541–750/767 CE), the Second Pandemic (including the Black Death, 1,346-18th century CE), and the Third Pandemic (c. 1,772–1,945 CE), which is believed to have originated in southwestern Yunnan Province, China, around 1772 and spread globally in the early 20th century (4). Currently, plague persists endemically within natural plague foci, where *Y. pestis* is maintained through complex cycles involving hosts, vectors, and the environment, enabling its long-term persistence (5). *Yersinia pestis* has been documented in at least 33 countries; over 200 mammalian species may be infected, mainly through soil contact or burrowing activities, and more than 30 flea species can transmit the bacterium among hosts (44). Human infection occurs via bites from infected ectoparasites, direct contact with infected animals, consumption of contaminated meat, or inhalation of respiratory droplets (4).

In China, 12 types of natural plague foci have been identified, classified by ecotype, landscape, hosts, vectors, and environmental features (6, 7). Yunnan Province harbors two major types: *Rattus tanezumi* focus and the *Apodemus chevrieri–Eothenomys miletus* focus. These are the most active foci in China (8) and accounted for approximately 60% of human plague cases in China from 1986 to 2005. Each focus has a unique ecological environment, including geography, climate, hosts, vectors, landscape, and soil properties, which regulate shifts between quiescent and active periods (9). Environmental persistence of *Y. pestis* has been proposed as a key mechanism for inter-epizootic maintenance, but direct evidence remains limited (10).

However, the mechanisms underlying sudden re-emergence, prolonged quiescence, and geographic boundaries of plague foci remain poorly understood. Studies over centuries have shown that plague dynamics are closely linked to local ecological stability (9). The ability of *Y. pestis* to persist in soil further suggests that soil physicochemical properties may strongly influence its survival. Nevertheless, studies on how soil characteristics affect plague dynamics remain limited.

As a fundamental component of plague foci, soil supports microbial, rodent, and flea communities and is closely related to the quiescence and re-emergence of plague. Soil chemistry may promote or inhibit epizootics by influencing the survival of *Y. pestis* (11), flea and host persistence and reproduction, as well as human activities, thereby reshaping the host-vector-environment balance. Soil pH, soil electrical conductivity (SEC), and soil organic matter (SOM) are key physicochemical indicators that reflect soil quality and influence microbial community structure and diversity. Soil metal elements may also contribute to the long-term maintenance of *Y. pestis* in plague foci (12). However, the specific relationships between soil properties and plague occurrence remain insufficiently understood and require further investigation. In recent years, sporadic human cases and animal epizootics in Yunnan indicate that the ecological balance involving *Y. pestis* has been disturbed, suggesting potential risk of renewed activity. Therefore, active monitoring of environmental changes in historical foci—including soil acidity, salinity, aridity, and mineral content—is urgently needed (13).

This study aimed to characterize surface soil properties in historical commensal rodent plague foci in Yunnan Province and compare them with matched non-plague villages. The objectives were to reveal differences in soil pH, SEC, SOM, and metal elements (Fe, Ca, Ti, Co, Cu, Ni, V) across regions, land use types, and plague history groups; to explore their correlations; and to provide baseline data for understanding soil environments during plague quiescence. This study addresses only the environmental component of a One Health framework and is intended to complement host/vector investigations. The findings may support the development of integrated plague surveillance and control strategies.

## 2 Materials and methods

### 2.1 Sample sites

**Figure 1:**
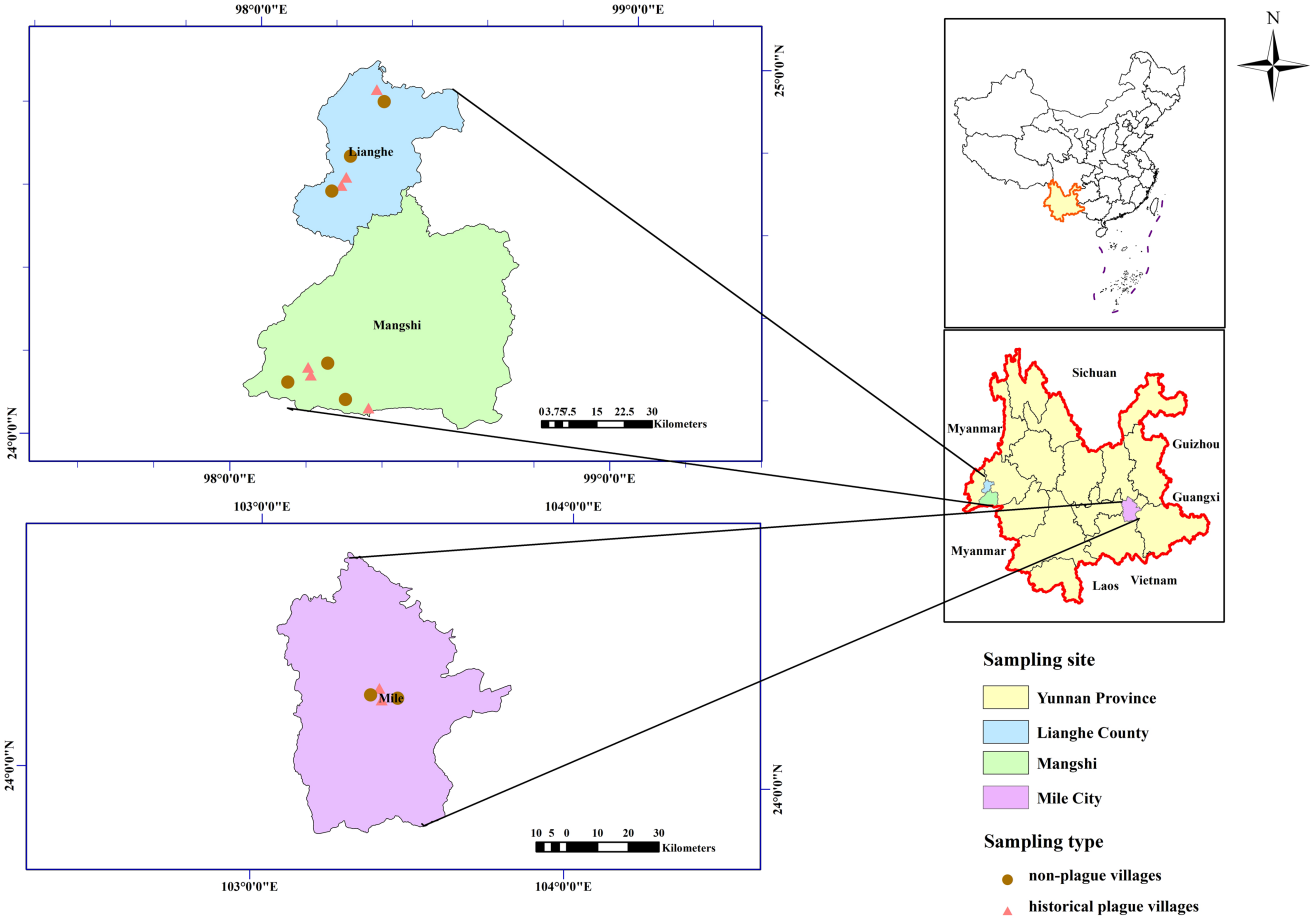
Geographic locations of historical plague villages and non-plague control villages in three study areas, Yunnan Province, China.

This was a cross-sectional study conducted in typical commensal rodent plague foci in Yunnan Province from July to August 2019. A total of 16 natural villages were included, located in Mile, Lianghe, and Mangshi counties. The geographic distribution of sampling sites is shown in Figure 1. A paired study design was applied: 8 historical plague villages (with at least 2 recorded plague outbreaks), 8 non-plague control villages located 5–10 km from corresponding plague villages, matched for population size, household number, economic status, ethnic customs, and geographic environment to minimize selection bias. Detailed sampling information is listed in Table 1.

**Table 1:** Summary of soil sampling in epidemic foci.

County	Village	Type	Code	Soil sample size
Mile	Asuobai	Plague	A	14
	Yangjie	Non-plague	B	22
	Suya	Plague	C	14
	Dahongtang	Non-plague	D	22
Mangshi	Wonglong	Plague	E	10
	Bangma	Non-plague	F	17
	Laxiang	Plague	G	18
	Nanbang	Non-plague	H	13
	Zhehuan	Plague	I	16
	Dongxiang	Non-plague	J	13
Lianghe	Xiaomengwu	Plague	K	11
	Bingsai	Non-plague	L	12
	Xiaowan	Plague	M	13
	Mangmeng	Non-plague	N	12
	Nameng	Plague	O	12
	Yangliuhe	Non-plague	P	11

Soil samples were collected from four directions (east, south, west, north) around residential areas in each village. Sampling points were positioned at rat tracks and rodent paths to represent soil environments closely associated with rodent activity. At each sampling point, after removing vegetation, weeds, stones, and loose surface soil, soil samples were collected using a wooden spatula at approximately 10 cm below the ground surface. The samples were then placed into sealed plastic bags, labeled uniquely, and stored at 4 °C until laboratory analysis. Soil texture was determined by standard field morphological assessment, based on soil feel, cohesion, and moisture retention characteristics, and classified into three types: sandy soil, loam, and sandy loam. Land-use type and other field parameters were recorded during the survey.

### 2.2 Soil sample preparation

Air-dried soil samples were crushed and ground using wooden tools, sieved through a 10-mm nylon sieve. Each soil sample was divided into two portions, one portion was used for the determination of pH, SEC, and dry matter content for subsequent metal concentration calculation. The remaining portion was further sieved through a 60-mm nylon sieve and then split again into two subsamples. One subsample was used for SOM determination, and the other was passed through a 100 mm nylon sieve for metal analysis.

### 2.3 Soil physicochemical and metal element analysis

Soil samples were processed according to the Environmental Protection Standards of the People’s Republic of China.

#### 2.3.1 Analysis of soil pH, SEC, and SOM

Soil pH and SEC were measured in a 1:2.5 (w/v) soil-deionized water suspension after 30 min of equilibration with occasional stirring, using a calibrated pH meter (PHS-3E, Shanghai Yi Electrical) and a conductivity meter (ST3100c/F, Ohaus), respectively, in accordance with the national standards HJ 962–2018 (pH) and HJ 802–2016 (SEC).

SOM content was determined by the potassium dichromate oxidation (Walkley-Black) method with external heating, in accordance with standard NY/T 1121.6–2006. In this method, soil organic carbon is oxidized by potassium dichromate under heated conditions. Excess dichromate is then titrated with ferrous sulfate to calculate the organic carbon content, which is subsequently converted to SOM using a conversion factor of 1.724. Results are reported in g/kg.

#### 2.3.2 Determination of soil dry matter content

Soil dry matter content was determined following standard HJ 613–2011. Briefly, aliquots of air-dried samples were dried in a moisture analyzer at 105 °C for 10 min. The obtained dry matter values were used to correct the concentrations of soil metal elements for the moisture content of the air-dried samples.

#### 2.3.3 Analysis of metal elements

##### 2.3.3.1 Digestion

Approximately 0.2000 g of finely ground soil (<100 mesh) was accurately weighed into a polytetrafluoroethylene (PTFE) digestion vessel in a fume hood. A mixed acid digestion system (6 mL concentrated HNO_3_, 3 mL concentrated HCl, 1 mL concentrated HF) was added to each vessel, and the mixture was gently swirled for homogenization. After hermetic sealing, microwave-assisted digestion was conducted using a Multiwave PRO system (Anton Paar, Austria) in accordance with the national standards HJ/T 166–2004 and HJ 832–2017, with the specific temperature program detailed in Table 2. Following digestion, 6 mL of saturated boric acid solution was added to complex excess fluoride ions and eliminate matrix interference; digested solutions were then heated at 150 °C for approximately 200 min to evaporate acids until the residual volume was reduced to ~1 mL. The inner walls of the digestion vessels and lids were rinsed at least three times with 3% (v/v) HNO₃ to ensure complete sample transfer. All rinsates and the digested solution were combined, transferred into 50 mL polypropylene centrifuge tubes, and diluted to 50 mL with 3% (v/v) HNO_3_. Final solutions were filtered through slow quantitative filter paper prior to instrumental analysis.

**Table 2:** Operating procedure of microwave digestion instrument.

Step	Procedure	Time(min)	Temperature(°C)	Fan level
1	Temperature rising	10	Room temperature →120	1
2	Hold	5	120	1
3	Temperature rising	10	120 → 190	1
4	Hold	30	190	1
5	Cooling	8	190 → 70	3

##### 2.3.3.2 Instrumental analysis

The concentrations of seven metal elements (Fe, Ca, Ti, Co, Cu, Ni, V) in filtered digestates were quantified using inductively coupled plasma optical emission spectrometry (ICP-OES; Optima 8000DV, PerkinElmer). The instrumental operating parameters are summarized in Table 3. To ensure analytical stability and minimize instrumental drift, a 30-s sample introduction delay was applied, and each sample was analyzed with two replicate injections.

**Table 3:** Operating parameter of ICP-OES.

Item	Setting
RF power	1.3 kW
Carrier gas	Argon
Plasma flow rate	15 L/min
Auxiliary gas flow rate	0.2 L/min
Nebulizer gas flow rate	0.55 L/min
Nebulizer back pressure	264 kPa
Flush pump flow rate	1.5 L/min
Pump stabilization time	30 s
Instrument repeats	2

##### 2.3.3.3 Calibration and quality assurance/quality control

A multi-element calibration standard (NCS 148664, NCS Testing Technology Co., Ltd.) was serially diluted with 3% HNO₃ to prepare the working standard solutions. All calibration curves exhibited excellent linearity, with correlation coefficients (r) > 0.9995. Method detection limits (MDL) and limits of quantitation (LOQ) were calculated as three and 10 times the standard deviation (SD) of 11 procedural blank measurements, respectively; detailed linear equations, MDL and LOQ for all elements are listed in Table 4.

**Table 4:** Curve correlation coefficient and detection limit.

Element	Linear equation	Correlation coefficient	SD (mg/L)	Method detection limit (mg/L)	Limit of quantitation(mg/L)
Fe	*y* = 51,370 *x* + 57340.5	0.9998	0.0010	0.0030	0.0100
Ca	*y* = 50,140 *x* + 18257.4	0.9999	0.0017	0.0051	0.0169
Ti	*y* = 392,700 *x* - 138431.1	0.9998	0.0022	0.0065	0.0218
Co	*y* = 21,720 *x* + 4609.4	0.9995	0.0010	0.0031	0.0102
Cu	*y* = 53,920 *x* + 10674.6	0.9996	0.0007	0.0020	0.0066
Ni	*y* = 19,360 *x* + 3530.0	0.9996	0.0005	0.0014	0.0045
V	*y* = 49,950 *x* + 3272.5	0.9999	0.0007	0.0021	0.0071

The accuracy and precision of the entire analytical procedure were validated by analyzing the certified reference material GBW07403a (GSS-3a) in parallel (*n* = 6). For all target elements, recoveries relative to the certified values ranged from 89.7 to 102.9%, with relative standard deviations (RSD) below 6.7%. Spike recovery tests performed on representative sample matrices yielded recoveries of 85–105%, meeting standard analytical quality requirements; detailed data are listed in Table 5. Procedural blanks and duplicate samples were included in each digestion batch to monitor potential contamination and assess method reproducibility.

**Table 5:** Precision and accuracy of the determination method.

Elements	Reference value (mg/L)	Mean measured value (mg/L)	RSD (%)	Spiked amount (mg/L)	Mean measured value after spiking (mg/L)	Recovery (%)
Fe	73.640	75.991	3.19	30	104.523	102.94
Ca	24.000	22.671	5.54	30	50.911	89.70
Ti	9.120	8.517	6.61	15	22.855	91.57
Co	0.028	0.029	4.27	3	2.942	97.13
Cu	0.054	0.050	5.96	3	2.964	97.03
Ni	0.060	0.057	4.63	3	3.106	101.55
V	0.180	0.185	2.77	3	2.974	93.12

### 2.4 Statistical analysis

All data were recorded and arranged with Microsoft Excel 2019 to establish a database of soil characteristics of plague foci in Yunnan Province. SPSS 26.0 was used for statistical analysis. Prior to the analysis, data on soil properties were explored to confirm whether the normality and homogeneity of the variance were satisfied by the Shapiro test and the Levene’s test, respectively. Soil characteristics were presented as mean and standard deviation for normally distributed data, and median and interquartile range [*M (P_25_, P_75_)*] for non-normal data. The Wilcoxon signed rank test, Kruskal Wallis *H* test and Mann–Whitney *U* test were used to analyze the differences in soil characteristics across regions, land use types, and historical plague status. Bonferroni correction and Mann–Whitney *U* test were used for multiple comparisons. Spearman’s rank correlation was used to analyze the correlations among soil properties.

To reduce dimensionality and multicollinearity among soil variables, principal component analysis (PCA) was performed based on 10 soil indicators. The Kaiser-Meyer-Olkin (KMO) test and Bartlett’s test of sphericity were used to evaluate data adequacy for PCA. Components with eigenvalues > 1 were retained. The extracted principal components, together with soil texture and land-use type, were then included as independent variables in a binary logistic regression model to identify key factors associated with historical plague village status. *p* < 0.05 was considered statistically significant.

## 3 Results

A total of 230 samples were collected in this study, including 108 from historical plague villages and 122 were from non-plague villages, of which 72 samples were obtained from Mile, 87 from Mangshi, 71 from Lianghe. The samples covered three land use types (arable land, forest land, grassland) and three soil textures (sandy soil, loam, sandy loam).

### 3.1 Soil characteristics of plague foci

The pH of the samples in this study ranged from 4.05 to 8.37, the median was 5.82, the soil electric conductivity (SEC) ranged from 33.3 μS/cm to 3,870 μS/cm, the median was 166.51 μS/cm, the soil organic matter (SOM) varied from 0.85 g/kg to 346.095 g/kg, whose median was 21.139 g/kg. The results of seven soils metal (Fe, Ti, Ca, V, Ni, Cu, Co) were 53592.11 mg/kg (13742.76–198628.02 mg/kg), 5360.89 mg/kg (1257.48–28110.21 mg/kg), 1733.82 mg/kg (0.00–102324.35 mg/kg), 146.80 mg/kg (25.31–653.29 mg/kg), 46.38 mg/kg (0.00–397.58 mg/kg), 39.87 mg/kg (1.90–168.09 mg/kg), 26.49 mg/kg (8.69–90.92 mg/kg), respectively. Fe was the most abundant, while Ni, Cu and Co were relatively low, with median values below 100 mg/kg.

Compared with national soil environmental background values, six metals (Fe, Ti, Co, Cu, Ni, V) were significantly elevated (all *p* < 0.001). The degree of exceedance relative to background values ranked as follows: Co (128.36%) > Cu (92.61%) > V (91.15%) > Ni (86.26%) > Fe (80.44%) > Ti (40.71%), while the median Ca content was much lower than the background, with a median of 18.64% and a mean of 60.79% of the background. The soil characteristics of the plague foci were shown in Table 6.

**Table 6:** Overall distribution of soil characteristics in the study regions (*n* = 230).

Item	Mean	Median	Standard deviation	Minimum	Maximum	Background value		
						Mean	Median	P^a^
pH	5.93	5.82	1.08	4.05	8.37	NA	NA	NA
SEC(μS/cm)	244.02	166.51	345.18	33.30	3870.00	NA	NA	NA
SOM(g/kg)	25.79	21.139	27.47	0.85	346.09	NA	NA	NA
Fe(mg/kg)	63548.62	53592.11	35036.86	13742.76	198628.02	29400.00	29700.00	<0.001*
Ca(mg/kg)	9361.09	1733.82	18406.01	0.00	102324.35	15400.00	9300.00	<0.001*
Ti(mg/kg)	7515.98	5360.89	5425.52	1257.48	28110.21	3800.00	3810.00	<0.001*
Co(mg/kg)	30.48	26.49	14.94	8.69	90.92	12.70	11.60	<0.001*
Cu(mg/kg)	46.58	39.87	31.18	1.90	168.09	22.60	20.70	<0.001*
Ni(mg/kg)	56.64	46.38	51.19	0.00	397.58	26.90	24.90	<0.001*
V(mg/kg)	201.89	146.80	138.29	25.31	653.29	82.40	76.80	<0.001*

^a^*p*-values were obtained from the Wilcoxon signed-rank test, comparing measured soil characteristics with national soil background values. The significance level α was 0.05. *Represents *p* < 0.001. NA represents no available data.

### 3.2 Comparison of soil characteristics between historical plague villages and non-plague control villages

Descriptive statistics of the soil characteristics in this study are presented in Table 7. Overall, Ni and V concentrations were significantly lower in historical plague villages than in non-plague villages (both *p* < 0.05), whereas no significant differences were observed for the other variables. However, patterns varied across regions. In Mile County, historical plague villages have lower SEC, SOM, Fe, Ti, Cu, Ni, and V than the non-plague villages, while differences in pH, Ca and Co content were non-significant. In Mangshi County, historical plague villages had higher SOM, content of Ti, Co and Cu than the non-plague epidemic villages, while differences in the pH, SEC, Fe, Ca, Ni, and V were non-significant. In Lianghe County, historical plague villages had higher SEC and content Ti content than non-plague control villages, while differences in pH, SOM, Fe, Ca, Co, Cu, Ni, and V were non-significant.

**Table 7:** Comparison of soil characteristics between historical plague villages and neighboring non-plague control villages [*M*(*P_25_, P_75_*)].

Region	Type	pH	SEC(μS/cm)	SOM(g/kg)	Fe(mg/kg)	Ca(mg/kg)	Ti(mg/kg)	Co(mg/kg)	Cu(mg/kg)	Ni(mg/kg)	V(mg/kg)
The whole	Plague (*n* = 108)	5.93 (5.16, 6.78)	152.00 (97.78, 288.50)	21.16 (13.59, 30.22)	51700.52 (41136.50, 65778.03)	2665.28 (814.93, 9078.13)	5595.78 (4726.40, 6309.04)	27.17 (22.46, 33.75)	38.90 (24.75, 53.15)	39.93 (19.76, 64.85)	137.69 (120.16, 173.46)
	Control (*n* = 122)	5.70 (4.92, 6.92)	172.20 (101.18, 279.75)	21.35 (13.38, 30.37)	54420.20 (41929.80, 88013.68)	1362.11 (278.96, 11859.03)	5012.64 (4103.31, 9401.22)	25.26 (16.61, 46.56)	41.54 (20.84, 71.46)	52.12 (25.47, 88.69)	169.93 (111.33, 338.90)
	Z (*P*_Z_)	−0.993 (0.321)	−0.202 (0.840)	−0.238 (0.812)	−1.305 (0.192)	−1.509 (0.131)	−0.838 (0.402)	−1.125 (0.261)	−0.757 (0.449)	−2.003 (0.045*)	−2.617 (0.009**)
Mile	Plague (*n* = 28)	6.88 (5.87, 7.47)	154.30 (100.00, 306.25)	14.81 (9.25, 20.56)	76490.06 (33267.42, 100793.01)	2252.49 (0.00, 27946.20)	9768.10 (4754.00, 17843.37)	45.21 (22.97, 50.77)	67.03 (27.90, 87.51)	55.61 (37.08, 92.07)	243.55 (108.14, 328.95)
	Control (*n* = 44)	6.15 (5.49, 7.32)	252.50 (167.08, 405.00)	25.72 (16.70, 39.97)	118749.61 (57562.51, 141212.19)	1838.89 (0.00, 27893.78)	15910.38 (7561.12, 19260.26)	48.61 (34.71, 56.41)	84.56 (61.32, 92.18)	92.31 (69.10, 118.80)	416.79 (240.59, 480.97)
	Z (*P*_Z_)	−0.884 (0.377)	−2.819(0.005**)	−3.893 (<0.001**)	−2.738 (0.006**)	−0.047 (0.962)	−2.299 (0.022*)	−1.779 (0.075)	−2.743 (0.006**)	−3.650 (<0.001**)	−3.535 (<0.001**)
Data are presented as median [interquartile range, M(P_25_, P_75_)]. Z and *P*-values were derived from the Mann–Whitney U test. *Represents *p* < 0.05, **represents *p* < 0.01.											

Region	Type	pH	SEC(μS/cm)	SOM(g/kg)	Fe(mg/kg)	Ca(mg/kg)	Ti(mg/kg)	Co(mg/kg)	Cu(mg/kg)	Ni(mg/kg)	V(mg/kg)
Mangshi	Plague (*n* = 44)	5.83 (5.07, 6.61)	141.65 (93.73, 294.00)	23.13 (20.24, 33.70)	45717.08 (41978.46, 55518.61)	1116.37 (638.90, 2730.43)	5113.03 (4726.03, 5797.81)	25.86 (23.20, 29.18)	40.09 (36.06, 46.63)	40.30 (19.76, 62.93)	145.84 (127.49, 168.75)
	Control (*n* = 43)	5.28 (4.47, 6.60)	170.70 (98.50, 217.00)	19.12 (13.35, 23.90)	45467.26 (34991.96, 53242.74)	506.40 (328.19, 1995.77)	4230.54 (3580.58, 4776.72)	16.58 (13.77, 21.36)	36.33 (27.90, 44.50)	29.71 (19.15, 50.38)	136.32 (105.08, 174.44)
	Z (*P*_Z_)	−1.749 (0.080)	−0.539 (0.590)	−2.793 (0.005*)	−1.435 (0.151)	−1.817 (0.069)	−4.865 (0.001**)	−5.815 (<0.001**)	−2.411 (0.016*)	−1.562 (0.118)	−0.908 (0.364)
Lianghe	Plague (*n* = 36)	5.65 (5.06, 6.04)	150.65 (88.48, 248.25)	24.78 (12.41, 30.68)	58300.12 (42750.28, 67848.86)	6911.04 (3566.13, 13158.31)	5649.33 (4724.40, 6235.00)	27.06 (20.56, 31.29)	18.14 (13.16, 44.37)	23.00 (12.26, 50.40)	128.12 (94.82, 142.22)
	Control (*n* = 35)	5.14 (4.93, 6.16)	118.30 (56.90, 170.70)	19.64 (7.28, 27.39)	53677.23 (33823.03, 66307.50)	2019.87 (507.27, 13254.91)	4587.37 (3471.03, 6061.93)	23.88 (16.91, 27.86)	16.45 (12.89, 24.15)	29.10 (13.24, 59.93)	112.56 (74.39, 163.72)
	Z(*P*_Z_)	−0.483 (0.629)	−2.363 (0.018*)	−1.576 (0.115)	−0.863 (0.388)	−1.794 (0.073)	−2.611 (0.009**)	−1.558 (0.119)	−1.139 (0.255)	−1.018 (0.309)	−0.794 (0.427)

Z is the statistic tested by Mann–Whitney U test, significance level α = 0.05, *represent *P* < 0.05, **represent *P* < 0.01.

### 3.3 Comparison of soil characteristics in different regions

**Figure 2:**
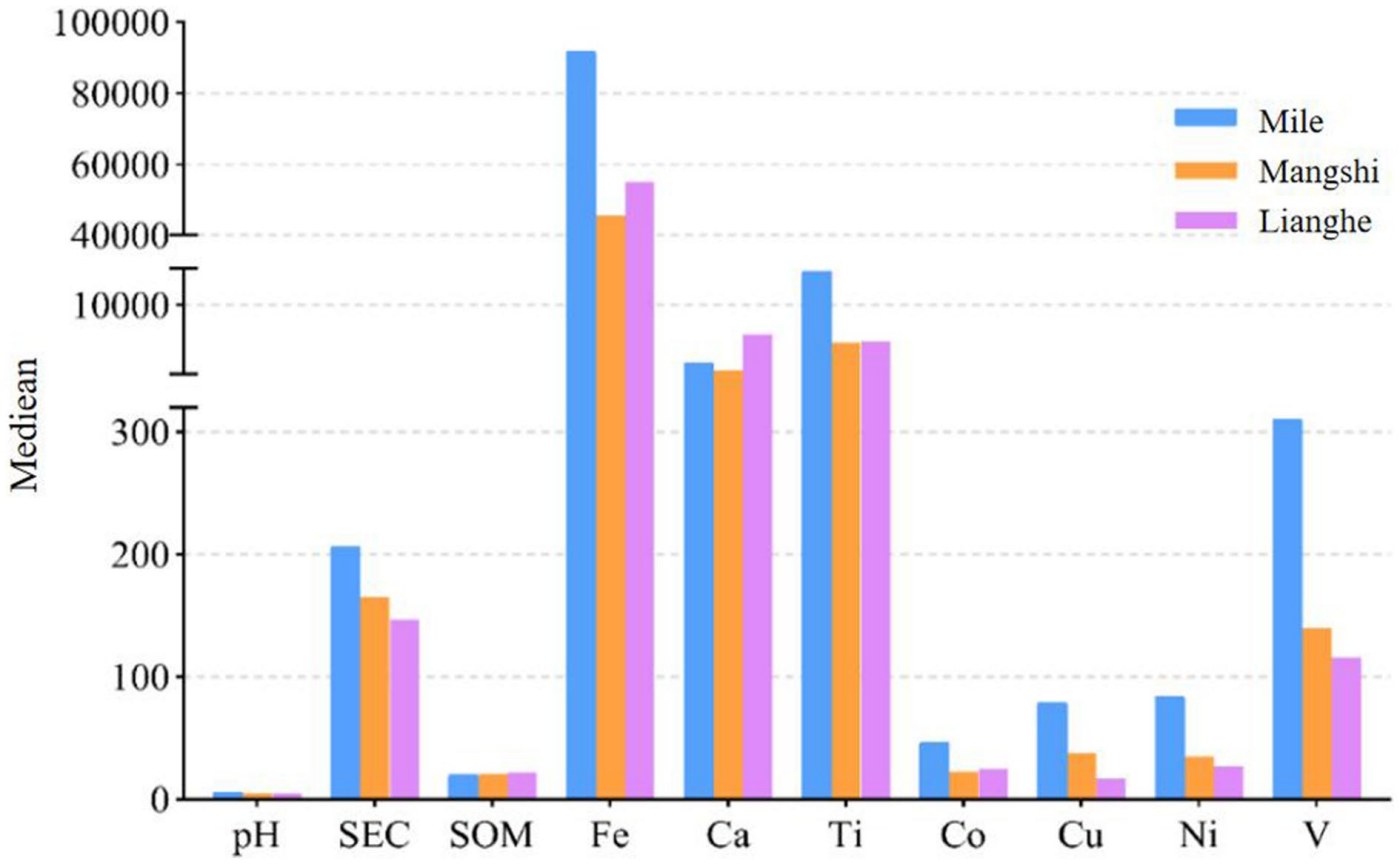
Distribution of soil properties of plague foci in three regions.

Descriptive statistics of soil characteristics in the three counties were presented in Figure 2 and Table 8. All characteristics differed significantly across regions (all *p* < 0.05) except SOM (*p* = 0.634). The median soil pH was 6.79, 5.57, 5.48 in Mile, Mangshi, Lianghe, respectively. There was a significant difference among the three regions, with Mile higher than Mangshi and Lianghe. The SEC medians of Mile, Mangshi, Lianghe were 207.5 μS/cm, 165.90 μS/cm, 147.10 μS/cm, respectively. There was a significant difference among the three regions, with Mile higher than Mangshi and Lianghe. The seven soil metals in the three regions were not all the same. For the soil metal Fe, the median sequence of the three regions is Mile > Lianghe > Mangshi. The sequences of Cu, V are Mile > Mangshi > Lianghe, and the contents of Co, Ni, and Ti were the highest in Mile, while Lianghe and Mangshi did not differ. For the soil metal Ca, the median value was highest in Lianghe, and there was no difference between Mile and Mangshi. *Post-hoc* comparisons confirmed that Mile had significantly higher pH, SEC, Fe, Ti, Co, Cu, Ni, and V than both Mangshi and Lianghe (Bonferroni-corrected *p* < 0.017).

**Table 8:** Comparison Soil characteristics among three regions [*M(P_25_, P_75_)*].

Region	pH	SEC(μS/cm)	SOM(g/kg)	Fe(mg/kg)	Ca(mg/kg)	Ti(mg/kg)	Co(mg/kg)	Cu(mg/kg)	Ni(mg/kg)	V(mg/kg)
Mile (*n* = 72)	6.79 (5.51, 7.39)	207.5 (136.3,367)	20.5 (13.4, 31.92)	91741.03 (51442.89, 128809.81)	2043.53 (0, 27946.2)	14678.78 (6279.84, 18788.70)	47.47 (32.35, 51.48)	79.94 (54.8, 90.22)	84.37 (55.53, 110.47)	310.1 (199.02, 460.19)
Mangshi (*n* = 87)	5.57 (4.82,6.60)	165.9 (97.7,284.00)	21.41 (15.27, 29.82)	45467.26 (39979.15,54001.47)	1013.26 (427.13,2423.98)	4740.25 (4189.12,5392.86)	23.06 (16.42,26.69)	38.17 (30.74,44.78)	35.89 (19.69,58.79)	140.41 (122.69,170.01)
Lianghe (*n* = 71)	5.48 (5.02,6.06)	147.1 (76.6,212)	22.05 (8.67, 30.02)	54892.67 (38490.9,66537.14)	5896.41 (1629.65,13254.91)	5049.57 (4342.12,6097.93)	25.32 (18.9,30.45)	17.55 (13.04,38.65)	27.59 (12.84,57.08)	116.77 (91.48,146.42)
H (P_H_)	27.55 (<0.001*)	17.717 (<0.001*)	0.911 (0.634)	43.21 (<0.001*)	20.662 (<0.001*)	82.203 (<0.001*)	88.858 (<0.001*)	98.781 (<0.001*)	69.786 (<0.001*)	74.194 (<0.001*)
Z^1^ (P_Z_^1^)	−4.249 (<0.001**)	−2.888 (0.004**)	–	−6.031 (<0.001**)	−0.81 (0.418)	−8.294 (<0.001**)	−8.844 (<0.001**)	−7.839 (<0.001**)	−7.126 (<0.001**)	−6.602 (<0.001**)
Z^2^ (P_Z_^2^)	−4.892 (<0.001**)	−3.949 (<0.001**)	–	−4.506 (<0.001**)	−1.595 (0.111)	−7.235 (<0.001**)	−7.082 (<0.001**)	−8.055 (<0.001**)	−7.300 (<0.001**)	−7.558 (<0.001**)
Z^3^ (P_Z_^3^)	−0.486 (0.627)	−1.825 (0.068)	–	−2.938 (0.003**)	−5.462 (<0.001**)	−1.777 (0.075)	−2.281 (0.023)	−5.516 (<0.001**)	−1.526 (0.127)	−3.744 (<0.001**)

H is the test statistic from the Kruskal-Wallis H test, with a significance level α = 0.05. *Represents *p* < 0.05. Z is the test statistic from the Mann–Whitney U test, with a Bonferroni-corrected significance level α’ = 0.017. **represents *p* < 0.017. 1 = Mile vs. Mangshi; 2 = Mile vs. Lianghe; 3 = Mangshi vs. Lianghe.

### 3.4 Comparison of soil characteristics among different land use types

The soil sample sources for this study include three land use types: arable land, woodland and grassland, and the measurements and statistical analysis of the soil characteristics were shown in Table 9. For the SEC, Fe, Ti, and Co, there was no statistically significant difference among the three types. Among the three land use types, grassland soil displayed higher pH value (6.02) than woodland (5.47), lower SOM value (16.02 g/kg) than cultivated land (22.43 g/kg)and woodland(23.53 g/kg), lower Cu value (36.19 mg/kg) and V value (136.75 mg/kg) than woodland(45.74 mg/kg, 167.69 mg/kg, respectively), While cultivated land displayed the lower Ni value (38.34 mg/kg) than woodland (54.69 mg/kg) and grass land (53.36 mg/kg), woodland displayed the lower Ca value (600.44 mg/kg) than cultivated land (1973.09 mg/kg) and grass land (3054.44 mg/kg).

**Table 9:** Descriptive statistics and difference analysis for Soil characteristics among three land use types [*M (P_25_, P_75_)*].

Type	pH	SEC(μS/cm)	SOM(g/kg)	Fe(mg/kg)	Ca(mg/kg)	Ti(mg/kg)	Co(mg/kg)	Cu(mg/kg)	Ni(mg/kg)	V(mg/kg)
Cultivated land (*n* = 98)	5.94 (5.09, 6.55)	183.65 (120.68, 290.50)	22.43 (16.10, 30.43)	52707.61 (38364.55, 66587.36)	1973.09 (575.04, 10955.06)	5349.76 (4361.06, 6551.96)	26.21 (19.53, 35.54)	39.57 (21.62, 54.50)	38.34 (15.75, 68.38)	151.65 (111.10, 208.72)
Woodland (*n* = 60)	5.47 (5.00, 6.20)	146.7 (91.48, 323.00)	23.53 (16.17, 33.99)	55628.53 (42988.83, 115560.15)	600.44 (157.09, 2410.07)	5812.20 (4589.87, 16887.37)	27.80 (19.99, 48.41)	45.74 (36.14, 83.82)	54.69 (25.96, 106.36)	167.69 (128.13, 430.62)
Grassland (*n* = 72)	6.02 (5.10, 7.41)	149.3 (85.73, 250.50)	16.02 (10.39, 27.61)	52712.18 (39752.66, 69147.94)	3054.64 (738.48, 13077.67)	4980.37 (4321.97, 6600.28)	25.76 (20.62, 32.89)	36.19 (18.82, 54.41)	53.36 (29.18, 75.37)	136.75 (102.39, 201.37)
H (*P_H_*)	6.497 (0.039*)	5.788 (0.055)	11.835 (0.003**)	3.933 (0.140)	16.45 (<0.001**)	4.449 (0.108)	1.27 (0.530)	7.456 (0.024*)	8.984 (0.011**)	9.478 (0.009**)
Z1 (P_Z_^1^)	−1.632 (0.103)	−1.471 (0.141)	−0.566 (0.571)	−1.834 (0.067)	−3.27 (<0.001**)	−1.379 (0.168)	−0.817 (0.414)	−2.202 (0.028)	−2.644 (0.008**)	−2.291 (0.022)
Z2 (P_Z_^2^)	−1.314 (0.189)	−2.321 (0.020)	−2.979 (0.003**)	−0.098 (0.922)	−1.019 (0.308)	−0.855 (0.393)	−0.358 (0.720)	−0.512 (0.608)	−2.253 (0.024*)	−1.083 (0.279)
Z3 (P_Z_^3^)	−2.431 (0.015**)	−0.736 (0.462)	−2.964 (0.003**)	−1.673 (0.094)	−3.806 (<0.001**)	−2.125 (0.034)	−1.115 (0.265)	−2.612 (0.009**)	−0.889 (0.374)	−2.948 (0.003**)

H is the test statistic from the Kruskal-Wallis H test, with a significance level α = 0.05. *represents *P* < 0.05, **represents *P* < 0.017 (Bonferroni-corrected significance level α’ = 0.017). Z is the test statistic from the Mann–Whitney U test: 1 = cultivated land vs. woodland; 2 = cultivated land vs. grassland; 3 = woodland vs. grassland.

### 3.5 Analysis of statistical correlations between soil properties

The results regarding statistical correlations between the soil properties were listed in Table 10. The SEC was positively correlated with pH (*r_s_ = 0.311, p <* 0.01), SOM (*r_s_ = 0.609, p <* 0.01), Ca element (*r_s_ = 0.243, p <* 0.01) and Cu elements (*r_s_ = 0.310, p <* 0.01); pH is positively correlated with Ca element (*r_s_ = 0.465, p <* 0.01) and Co element (*r_s_ = 0.160, p <* 0.05); SOM was positively correlated Cu element (*r_s_ = 0.257, p <* 0.01). The correlations between the seven metallic elements as a whole were relatively close. There was no significant correlation between Ca and Ti nor Co, and the Ca element is negatively correlated with Fe element (*r_s_ = −0.293, p <* 0.01), Cu element (*r_s_ = −0.246, p <* 0.01), Ni element (*r_s_ = −0.247, p <* 0.01) and V element (*r_s_ = −0.321, p <* 0.01), whereas the rest six metal elements were strongly positively correlation (*r_s_* ≥ *0.601, p <* 0.01).

**Table 10:** Spearman correlation matrix for soil characteristics (*n* = 230).

Characteristics	pH	SEC	SOM	Fe	Ca	Ti	Co	Cu	Ni	V
pH	1	0.311^**^	0.039	−0.076	0.465^**^	0.096	0.160^*^	0.113	0.123	0.025
SEC	0.311^**^	1	0.609^**^	−0.026	0.243^**^	0.087	0.084	0.310^**^	0.055	0.114
SOM	0.039	0.609^**^	1	0.005	0.086	0.111	0.101	0.257^**^	0.036	0.100
Fe	−0.076	−0.026	0.005	1	−0.293^**^	0.819^**^	0.771^**^	0.601^**^	0.661^**^	0.767^**^
Ca	0.465^**^	0.243^**^	0.086	−0.293^**^	1	−0.102	−0.047	−0.246^**^	−0.247^**^	−0.321^**^
Ti	0.096	0.087	0.111	0.819^**^	−0.102	1	0.854^**^	0.611^**^	0.657^**^	0.767^**^
Co	0.160^*^	0.084	0.101	0.771^**^	−0.047	0.854^**^	1	0.636^**^	0.735^**^	0.672^**^
Cu	0.113	0.310^**^	0.257^**^	0.601^**^	−0.246^**^	0.611^**^	0.636^**^	1	0.676^**^	0.765^**^
Ni	0.123	0.055	0.036	0.661^**^	−0.247^**^	0.657^**^	0.735^**^	0.676^**^	1	0.648^**^
V	0.025	0.114	0.100	0.767^**^	−0.321^**^	0.767^**^	0.672^**^	0.765^**^	0.648^**^	1

*Represenst *P* < 0.05, **represents *P* < 0.01.

### 3.6 Principal component analysis and binary logistic regression

Principal component analysis (PCA) was performed on 10 standardized soil indicators (pH, SEC, SOM, Fe, Ca, Ti, Co, Cu, Ni, V) to reduce dimensionality. The Kaiser–Meyer–Olkin (KMO) test yielded a value of 0.794, and Bartlett’s test of sphericity was statistically significant (*χ^2^* = 1560.54, *p* < 0.001), confirming the data were suitable for PCA. Four principal components were retained, explaining 83.72% of total variance (Table 11). The component matrix (Table 12) showed that PC1 was strongly positively loaded with Fe, Ti, Co, Cu, Ni, and V (all loadings > 0.68), representing a “heavy metal component”; PC2 was positively loaded with SEC and SOM (loadings > 0.80), representing a “soil physicochemical property component”; PC3 was positively loaded with pH and Ca (loadings > 0.64), representing a “soil pH-calcium component”; and PC4 was specifically loaded with Ni (loading = 0.694), representing a “nickel-specific component.”

**Table 11:** Principal component analysis eigenvalues and variance explained (*n* = 230).

Principal component	Eigenvalue	Variance explained (%)	Cumulative variance (%)
PC1	4.471	44.71	44.71
PC2	1.961	19.62	64.33
PC3	1.335	13.35	77.68
PC4	0.603	6.04	83.72

PCA was performed on 11 standardized soil indicators (dry matter, pH, SEC, SOM, Fe, Ca, Ti, Co, Cu, Ni, V). KMO = 0.794, Bartlett’s test of sphericity: χ^2^ = 1560.547, *P* < 0.001. Components with eigenvalue > 1 were retained (PC1–PC3), and PC4 was retained for model interpretability, with a total cumulative variance of 83.72%.

**Table 12:** Component matrix (factor loadings) for soil properties (*n* = 230).

Variable	PC1	PC2	PC3	PC4
pH (Zscore)	0.015	0.404	0.766	−0.107
SEC (Zscore)	0.104	0.855	−0.334	0.038
SOM (Zscore)	0.109	0.819	−0.376	0.024
Fe (Zscore)	0.928	−0.154	−0.063	−0.124
Ca (Zscore)	−0.135	0.509	0.650	0.008
Ti (Zscore)	0.907	−0.061	0.012	−0.223
Co (Zscore)	0.874	−0.032	0.188	0.016
Cu (Zscore)	0.873	0.298	−0.109	0.004
Ni (Zscore)	0.686	−0.096	0.142	0.694
V (Zscore)	0.866	−0.097	0.036	−0.209

Extraction method: Principal component analysis. All variables were standardized (Z-score) prior to analysis. Four components were extracted.

Binary logistic regression was conducted with the four PCs, land use type, and soil texture as independent variables, and historical plague village status (1 = plague village, 0 = non-plague village) as the dependent variable (reference: sandy soil, cultivated land). Only PC1 was independently associated with lower odds of historical plague occurrence (*β* = −0.276, *OR* = 0.759, 95% CI: 0.649–0.887, *p* = 0.001). Soil texture showed a significant overall association (Wald *χ^2^* = 7.304, *p* = 0.026): loam texture was associated with lower odds of historical plague occurrence compared with sandy soil (*β* = −0.889, *OR* = 0.411, 95% CI: 0.212–0.795, *p* = 0.008). Sandy loam and land use type showed no significant effects (all *p* > 0.05). Results are shown in Table 13.

**Table 13:** Binary logistic regression analysis for historical plague status (*n* = 230).

Variable	β (B)	S. E.	Wald χ^2^	df	*P*-value (Sig.)	Exp(B) (OR)	95% CI for Exp(B)	
							Lower	Upper
Categorical variables								
type overall	–	–	1.613	2	0.446	–	–	–
type(1) (woodland)	0.221	0.353	0.391	1	0.532	1.247	0.624	2.491
type(2) (grassland)	−0.262	0.331	0.629	1	0.428	0.769	0.402	1.471
texture (overall)	–	–	7.304	2	0.026	–	–	–
texture(1) (R-Loam)	−0.889	0.337	6.971	1	0.008	0.411	0.212	0.795
texture(2) (SR-Sandy Loam)	−0.897	0.578	2.412	1	0.120	0.408	0.131	1.265
Principal components (CF1–CF4)								
CF1 (PC1)	−0.276	0.080	11.934	1	0.001	0.759	0.649	0.887
CF2 (PC2)	0.185	0.141	1.717	1	0.190	1.203	0.912	1.587
CF3 (PC3)	−0.009	0.121	0.005	1	0.941	0.991	0.782	1.256
CF4 (PC4)	0.021	0.191	0.012	1	0.914	1.021	0.703	1.484
Constant	0.427	0.297	2.069	1	0.150	1.533	–	–

Dependent variable: Historical plague village status (1 = plague village, 0 = non-plague village). Reference categories: Cultivated land (for type), N-Sand (for texture). *P* < 0.05 was considered statistically significant.

## 4 Discussion

Plague is a rapidly spreading disease with a high fatality rate. Prevention focuses on minimizing exposure to *Y. pestis*, avoiding contact with infected fleas or carcasses, and using appropriate personal protective measures. In addition, active surveillance of indicator animals, susceptible rodents, environmental factors, and climate variability in plague foci is recommended as a good practice. Environmental factors, including soil-related variables, may substantially influence the transition between the dormant and active phases of plague (14). Some studies suggest that soil may be associated with the persistence of plague (15), although direct evidence remains limited. Associations between soil and plague foci may be related to effects on the pathogen, vectors, or hosts (16). As one of the ancient plague foci, the commensal rodent plague focus in Yunnan Province has remained active. Soil characteristics in this region are highly susceptible to human productive activities, which may indirectly affect the ecological balance of rodents, fleas, as well as the persistence of *Y. pestis*. All of these may be potential correlates of plague epidemics (17). Previous studies have shown that the distribution of metal elements is correlated with water and soil conditions in plague foci (18, 19). Previous studies have proposed, when the plague focus is in a resting state, *Y. pestis* may persist in the soil through parasitism associated with amoeba or non-parasitic survival (20), but this “soil-borne persistence” hypothesis remains unconfirmed in natural foci (21).

Soil characteristics constitute a key component of the plague ecosystem. Previous research indicates that soil properties can affect *Y. pestis* survival under controlled conditions (22) and influence flea and host ecology in the field, (23), highlighting the potential relevance of the soil environment. Our study provides an empirical, cross-sectional analysis of such associations in historical plague foci. Soil pH and SEC are key indicators of soil quality, reflecting soil acidity and salinity, respectively, while SOM indicates fertility (24). Our study shows that the soil in the foci was generally acidic, with median pH values ranging from 5.48 to 6.79, and exhibited mild salinization (median SEC: 166.51 μS/cm) according to the national classification standard (25). The near-neutral pH in Mile (6.79) aligns with previous local findings (26), whereas more acidic soils in Mangshi and Lianghe likely reflect differences in vegetation, soil composition, or agricultural practices. SOM (median 21.14 g/kg) was classified as Grade 3 (moderate fertility) according to the Second National Soil Survey (25, 27).

While *Y. pestis* exhibits some salt tolerance and saline-alkaline conditions have been hypothesized to influence plague ecology (28), this hypothesis requires validation in the specific context of our study areas. Soil salinization itself is a form of land degradation, often linked to human activities and environmental change (29). SOM content is influenced by soil type, texture, crop type, and human disturbance (24).

The distribution of natural plague foci has been linked to the concentration of certain metals in their environment. It is suggested that trace elements in soil environment may affect *Y. pestis* persistence and dynamics even in the absence of continuous “rodent-flea-rodent” transmission (11). Studies have confirmed that several metal elements in soil, such as Fe, Mg, and Se, are associated with the persistence of *Y. pestis* in the plague foci (28, 30). In additional studies, the researcher found that an excess or deficiency of some metal elements can affect the survival reproduction, virulence and activity of *Y. pestis in vitro*, influencing the functionality of bacterial protein transport systems and the synthesis of specific virulence factors, and ultimately enhances or reduces the ability of the pathogen to infect hosts (31–33). However, metal concentrations used in such experiments are orders of magnitude higher than those observed in this field study. Thus, these experimental findings cannot be directly extrapolated to our field conditions, and observed correlations do not imply a causal mechanistic link between soil metal and *Y. pestis* virulence.

Our study shows that soils in the three regions were enriched in Fe, Ti, and Ca, and had elevated Co, Cu, Ni, and V relative to national background values. These findings align with previous reports that plague foci are often located in Fe-rich areas (34). Li Hai-Rong reported that all rodent plague foci in southern China where plague epidemics occurred, had high in ferric iron in the soil (35). The current study is consistent with both lines of evidence. Soil properties differed notably between Mile and the other two counties, likely reflecting geographic and ecological differences between southern and southwestern Yunnan. Even between neighboring Mangshi and Lianghe, metals such as Fe, Ca, Cu, and V differed, indicating sensitivity to local environments and human activities. Expanded spatial sampling and further investigation of soil property determinants may improve understanding of soil-plague relationships.

In an early study, Zhangtao et al. (36) and his team reported that the plague could occur in rodent when the content of metal elements Fe, Ti, Co increased rapidly to three times of the normal level without clear reasons, or when the content of Cu, Ni and V suddenly dropped to 1/12 of normal level for unknown reasons while other metal ions remained normal level (12). In this study, historical plague villages exhibited a slightly lower levels of Fe, Cu, Ni, and V compared to non-plague control villages, while displaying higher concentrations of Ti, Co, and *Ca.* Significant differences in the Ni and V levels were observed between the two groups. In order to further clarify whether the difference was caused by different regions, we conducted a comparative analysis of soil metals across the three counties. The results demonstrated that in Mile county, the concentrations of Fe, Ti, Cu, Ni, and V were lower in historical plague villages than in non-plague control villages; in Mangshi, the concentrations of Ti, Co, and Cu were higher in historical plague villages than in non-plague control villages; in Lianghe county, the concentrations of Ti was higher in historical plague villages than in non-plague control villages. However, no significant differences were observed in the content of other soil metal elements between historical plague villages and non-plague control villages. These findings indicate that each plague focus has its own distinct ecological environment, and even within the same natural plague focus, the distribution of soil metal elements varies under different plague epidemic conditions. It is important to note that certain soil metal elements exhibit higher or lower levels in the historical plague villages compared to non-plague control villages across the region. This has led to speculation as to whether these variations in specific metallic elements are associated with the historical plague status observed in this study. Therefore, given Yunnan province’s highly complex terrain and abundant species diversity, it becomes imperative to closely monitor dynamic changes in soil metal element within different regions of plague focus (37), then study the correlation between soil metals and plague based on regional differences and considering multiple influencing factors, and improve the ability to predict future plagues outbreak.

The present study reveals significant correlation among the metal elements, with all except calcium exhibiting strong positive correlations. This finding aligns with previous investigations conducted in Gansu Huining (38) and Inner Mongolia (39). Positive or negative correlations among multiple metallic elements suggest that these elements exhibit synergistic and antagonistic effects in nature, which can affect the growth, reproduction, and ecological stability of organisms.

Among the three land use types examined in this study, woodland exhibited the highest levels of SOM, Cu, V, and Ni, while displaying the lowest values of pH and *Ca.* The spread of vector-borne diseases is influenced by two crucial factors: land utilization and human activities. These factors affect the spatial distribution of disease risk or incidence (40). Different land use patterns are associated with distinct soil characteristics, and may also be linked to variations in rodent population size, flea indices, and flea species composition in plague natural foci, as reported in previous studies (41–43). It should be emphasized that the present study did not measure rodent, flea, or human plague case data, so the above links between land use, rodent dynamics, and plague transmission are purely speculative hypotheses derived from the literature, not empirical observations from our research. The observed associations between land use type and soil characteristics in this study are correlational only, and do not imply a causal or mechanistic link between land use, soil properties, and plague transmission risk. Current policies such as returning farmland to forest or grassland, as well as urbanization construction projects such as community development and road construction have altered land use practices in China. Consequently, there has been a substantial change in ecological vegetation and a rapid shift in the rodent populations within a limited space over a short period of time. These disturbances may disrupt the natural ecological balance, potentially providing pathways for pathogen transmission to humans while increasing the risk associated with endemic pathogens.

In addition to univariate comparisons, principal component analysis (PCA) was used to reduce the dimensionality of 10 soil indicators, including pH, SEC, SOM, and multiple metal elements. Four principal components were extracted, explaining 83.72% of the total variance. PC1 was heavily loaded with Fe, Ti, Co, Cu, Ni, and V, representing a comprehensive heavy metal component; PC2 mainly reflected SEC and SOM, indicating soil physicochemical properties; PC3 was dominated by pH and Ca, representing soil acidity and calcium status; and PC4 was characterized by the independent variation of Ni. This classification helps to clarify the combined effects of multiple intercorrelated soil properties rather than interpreting each element in isolation. Binary logistic regression further demonstrated that PC1 (the comprehensive heavy metal component) was significantly associated with historical plague occurrence in the overall sample, with higher PC1 scores linked to lower odds of plague villages—consistent with the univariate finding that Ni and V were significantly lower in plague villages than in non-plague villages. Notably, this multivariate association is not consistent across the three study regions (Mile, Mangshi, Lianghe), as the direction and magnitude of metal differences varied substantially by county. Therefore, these findings do not support a universal, region-independent “plague soil signature” for Yunnan commensal rodent plague foci. Instead, soil metal profiles primarily reflect regional geological and anthropogenic backgrounds, and the observed PCA-derived association is a correlational pattern specific to our study sample. All observed associations are purely correlational and do not imply a causal or mechanistic link between soil metal levels and the historical village-level plague status we examined.

Soil texture showed a significant overall association with plague status, with loam soil linked to lower odds of historical plague villages compared with sandy soil. While laboratory and field studies suggest that soil physical properties may, in principle, influence vector habitat and pathogen persistence (22, 23), such mechanistic links remain speculative and were not investigated in our cross-sectional study. By contrast, habitat type (woodland, grassland, cultivated land) showed no independent effect in the multivariate model, suggesting that the influence of vegetation and land use may be largely mediated through soil properties rather than acting as an independent driver.

This study has several limitations, which we have attempted to address through supplementary analyses and clear contextualization, and we propose targeted improvements for future research. First, this was a cross-sectional study based on historical plague status, which inherently limits causal inference. To mitigate this limitation, we adopted a case–control study design, comparing soil characteristics between historical plague villages and non-plague control villages, to tentatively explore the potential associations between soil properties and plague occurrence, thereby reducing the bias caused by the cross-sectional design. Second, *Yersinia pestis* was not directly detected in soil in this study; therefore, conclusions regarding the role of soil in plague persistence should be interpreted with caution. Although our previous field surveys (2015–2016) in plague foci in Lianghe, Yulong, and Jianchuan counties in Yunnan Province also tested soil and water samples and failed to detect *Y. pestis* positivity (unpublished), the absence of positive findings does not rule out its potential environmental persistence under specific conditions. Accordingly, this study focuses on soil structural characteristics, and future studies incorporating molecular and microbiological approaches are needed to further elucidate the potential role of soil in plague ecology.

To elucidate the potential role of soil in plague outbreaks or epidemics, further studies should adopt a multidisciplinary and multidimensional approach. This includes long-term dynamic monitoring of spatial and temporal changes in soil metals, fluctuations in host animal populations and flea populations, assessment of *Y. pestis* presence and virulence, investigation into variations in plague transmission risk associated with changes in metallic element levels, and exploration of the contribution of soil to epidemic and quiescent patterns. Such findings are crucial for understanding the ecological correlates of plague and improving prevention and control strategies.

This study addresses only the environmental component of a One Health framework and is intended to complement host/vector investigations. A holistic understanding of plague persistence will require the integration of environmental, host, and vector data.

## 5 Conclusion

In this study, we characterized the surface soil properties in three commensal rodent plague foci in Yunnan. Soils were generally acidic, mildly salinized, and of moderate nutrient status (Grade 3). They were relatively enriched in Fe, Ti, and Ca, while Co, Cu, Ni, and V concentrations exceeded national background values. Principal component analysis (PCA) showed strong collinearity among multiple metal elements, whose spatial distribution mainly reflected regional environmental background differences rather than plague-specific signals. No consistent, universal soil signature associated with plague distribution was observed across regions. Binary logistic regression further indicated that the comprehensive heavy metal component (PC1) was significantly associated with historical plague occurrence, and soil texture was correlated with plague status, whereas habitat type showed no independent effect. These findings provide valuable baseline environmental data for local plague surveillance and further research on soil-epidemiology associations. Given the cross-sectional design and the lack of contemporaneous data on rodents, fleas, and *Yersinia pestis*, long-term dynamic monitoring, expanded spatial sampling, and integrated multi-domain data are needed to better clarify the role of soil in the persistence and activation of natural plague foci.

## Data Availability

The raw data supporting the conclusions of this article will be made available by the authors, without undue reservation.
